# Anomalous origin of coronary arteries with an interarterial course: pictorial essay

**DOI:** 10.1590/0100-3984.2017.0203

**Published:** 2019

**Authors:** Ana Flávia Pina Ferreira, Sharon Rosemberg, Daniel Simões Oliveira, José de Arimatéia Batista Araujo-Filho, Cesar Higa Nomura

**Affiliations:** 1 Instituto do Coração do Hospital das Clínicas da Faculdade de Medicina da Universidade de São Paulo (InCor/HC-FMUSP), São Paulo, SP, Brazil.; 2 Hospital Sírio-Libanês, São Paulo, SP, Brazil.

**Keywords:** Coronary vessel anomalies/complications, Coronary vessel anomalies/diagnostic imaging, Aorta, thoracic/abnormalities, Aorta, thoracic/diagnostic imaging, Anomalias dos vasos coronários/complicações, Anomalias dos vasos coronários/diagnóstico por imagem, Aorta torácica/anormalidades, Aorta torácica/diagnóstico por imagem

## Abstract

Coronary arteries originating from the contralateral (noncoronary) sinus and having an interarterial course, in which they run from the ascending aorta to the pulmonary trunk, is a potentially fatal anomaly. Computed tomography (CT) angiography facilitates the recognition and therapeutic planning of such anomalies because of its ability to acquire high-resolution images of the entire course of the coronary artery, as well as of the accompanying atherosclerotic involvement. The right coronary artery originating from the left coronary sinus is the most prevalent anomaly of this type and usually implies a better prognosis, the interarterial course being classified as "high" or "low", depending on whether it is above or below the level of the pulmonary valve, with consequent stratification of the risk and the treatment. However, it is known that there is a high risk of sudden death among patients with a left coronary artery of anomalous origin from the right sinus. In such cases, surgical treatment is recommended, regardless of whether there are symptoms or evidence of ischemia. Given the importance of those aspects, which can be identified on CT of the chest or CT angiography of the aorta, this pictorial essay aims to illustrate such anomalies to facilitate their recognition and description by radiologists who are not specialists in cardiac imaging.

## Abstract (pt)

O trajeto interarterial das artérias coronárias com origem em seio contralateral/não coronariano é uma anomalia potencialmente fatal caracterizada pelo trajeto das coronárias entre a aorta ascendente e o tronco da artéria pulmonar. A angiotomografia auxilia no reconhecimento e planejamento terapêutico dessas alterações, em virtude da sua capacidade em adquirir imagens de alta resolução de todo o trajeto coronariano, assim como do envolvimento aterosclerótico associado. A artéria coronária direita originada no seio coronariano esquerdo costuma ser mais prevalente e relacionada a um melhor prognóstico, sendo classificada em curso interarterial "alto" ou "baixo" de acordo com a altura do seu trajeto em relação à valva pulmonar, com consequente estratificação de risco e tratamento distintos. Sabe-se, entretanto, que há um elevado risco de morte súbita entre pacientes com artéria coronária esquerda de origem anômala a partir do seio direito, sendo recomendado tratamento cirúrgico, independentemente de sintomas ou evidência de isquemia. Em razão da importância desses achados que podem ser encontrados em exames de tomografia de tórax e angiotomografias de aorta, o presente ensaio tem por objetivo ilustrar as anomalias de trajeto das artérias coronárias, para facilitar seu reconhecimento e sua descrição por médicos radiologistas não especialistas em imagem cardíaca.

## INTRODUCTION

Anomalies of the coronary arteries are rare, present only in 1.3% of the population. However, approximately 20% are worrying as they potentially result in acute myocardial infarction, arrhythmia, or sudden death^(^^1^^)^. These anomalies can be categorized by origin, course, and termination. Among the anomalies related to the risk of sudden death, the following stand out: single coronary artery; ostial atresia; coronary artery originating from the pulmonary artery; large fistulae; and interarterial course with a coronary origin.

With advances in image acquisition techniques, multidetector computed tomography (CT) angiography has become a well-established method for evaluating coronary arteries (degree I recommendation and a B level of evidence, according to the Brazilian Society of Cardiology)^(^^2^^)^. Multidetector CT angiography is considered superior to conventional angiography for defining the origin and course of anomalous coronary branches^(^^3^^)^. With CT angiography, it is possible to acquire high-resolution images of the entire course of the coronary artery, as well as of potentially associated coronary atherosclerotic involvement, allowing potentially fatal alterations to be identified and facilitating therapeutic planning^(^^4^^-^^6^^)^.

The objective of this study is to show the importance of CT angiography in characterizing anomalous coronary arteries originating from the contralateral (noncoronary) sinus with an interarterial course, helping radiologists recognize and describe this important finding.

## NORMAL ANATOMY

**Figure 1:**
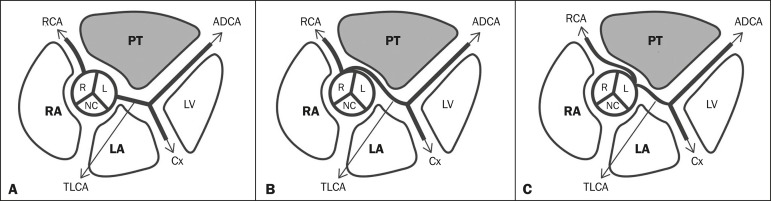
Illustration showing the usual origin of the coronary arteries (**A**), anomalous origin of the left coronary artery from the right coronary sinus, with an interarterial course (**B**), and anomalous origin of the right coronary artery from the left coronary sinus (**C**). RA, right atrium; LA, left atrium; PT, pulmonary trunk; LV, left ventricle; R, right coronary sinus; L, left coronary sinus; NC, noncoronary sinus; RCA, right coronary artery; TLCA, trunk of the left coronary artery; ADCA, anterior descending coronary artery; and Cx, circumflex coronary artery.

As depicted in Figure 1, the right coronary artery (RCA) arises from the anterior right coronary sinus, slightly below the left coronary artery (LCA), with a course immediately to the right of and posterior to the pulmonary artery, descending through the right atrioventricular sulcus toward the posterior interventricular septum^(^^3^^)^. The LCA originates from the left posterior coronary sinus, is 5-10 cm in length, and does not vary significantly in diameter. It courses to the left of and posterior to the pulmonary trunk, thereafter bifurcating into the anterior descending and circumflex coronary arteries^(^^3^^)^.

## INTERARTERIAL COURSE OF ANOMALOUS CORONARY ARTERIES ORIGINATING FROM THE CONTRALATERAL (NONCORONARY) SINUS

As can be seen in Figure 1, the left and right arteries can both originate from the contralateral sinus, constituting anomalies of origin and course. In a previous study involving patients undergoing angiography, it was observed that the RCA originated from the left sinus of Valsalva as a separate vessel or as a branch of a single coronary artery in 0.03-0.17% of the patients^(^^3^^)^. The LCA was also observed to originate from the right sinus of Valsalva as a separate vessel or single branch of the coronary artery in 0.09-0.11% of the cases, and an interarterial course was seen in more than 75% of the patients with that anomaly^(^^3^^)^.

Although the RCA originating from the left coronary sinus is more prevalent and related to a better prognosis, it is known to be a high risk of sudden death among patients with LCA of anomalous origin from the right sinus^(^^7^^)^. In the latter cases, the therapeutic decision-making process is complex and still controversial, given the importance of associated symptoms, ischemia, as detected by functional methods, and, more recently, the type of interarterial course found.

## RCA

**Figure 2:**
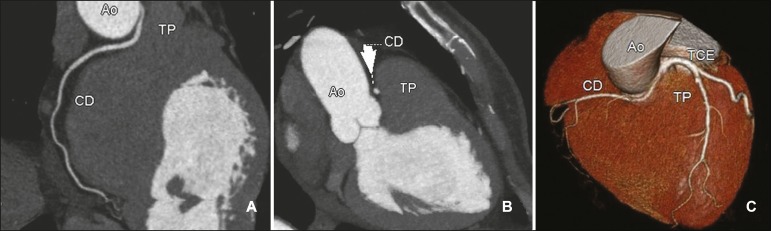
Contrast-enhanced CT. Oblique reconstructions (**A,B**) and a three-dimensional reconstruction (**C**) showing anomalous origin of the right coronary artery, with a course from the aorta to the pulmonary trunk. The image in (**B**) also shows the spot sign (arrow). CD, right coronary artery; Ao, aorta; TP, pulmonary trunk; TCE, trunk of the left coronary artery.

**Figure 3:**
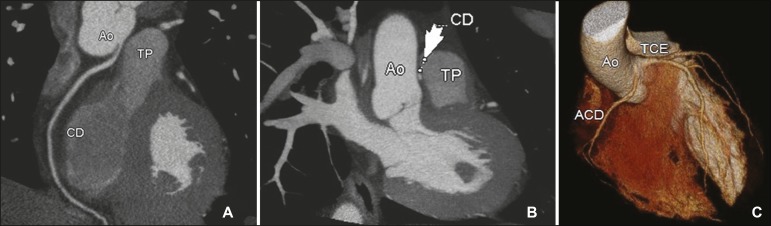
Contrast-enhanced CT. Oblique reconstructions (**A,B**) and a three-dimensional reconstruction (**C**) showing anomalous origin of the right coronary artery from the left coronary sinus, with a course from the aorta to the pulmonary trunk. CD/ACD, right coronary artery; Ao, aorta; TP, pulmonary trunk; TCE, trunk of the left coronary artery.

**Figure 4:**
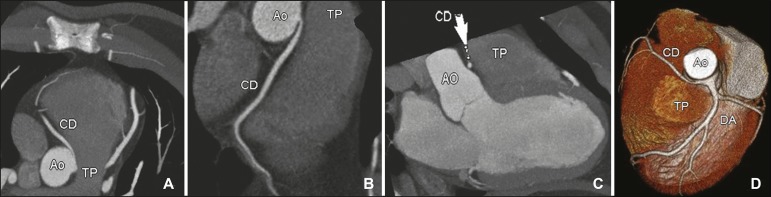
Contrast-enhanced axial CT (**A**), oblique reconstructions (**B,C**), and a three-dimensional reconstruction (**D**) showing the anomalous interarterial course of an right coronary artery originating from the contralateral (left) sinus and its course from the aorta to the pulmonary trunk. CD, right coronary artery; Ao, aorta; TP, pulmonary trunk; DA, anterior descending coronary artery.

Figures 2, 3, and 4 show examples of anomalous origin of the RCA. The most common course of an anomalous RCA that arises from the sinus of Valsalva is interarterial (between the ascending aorta and the pulmonary trunk). This variant is associated with sudden death in up to 30% of patients^(^^3^^)^. In such cases, it is believed that the dilation of the aorta during physical exercise can narrow the anomalous ostium, reducing blood flow in the coronary artery predisposing to ischemic myocardial alterations^(^^7^^)^.

**Figure 5:**
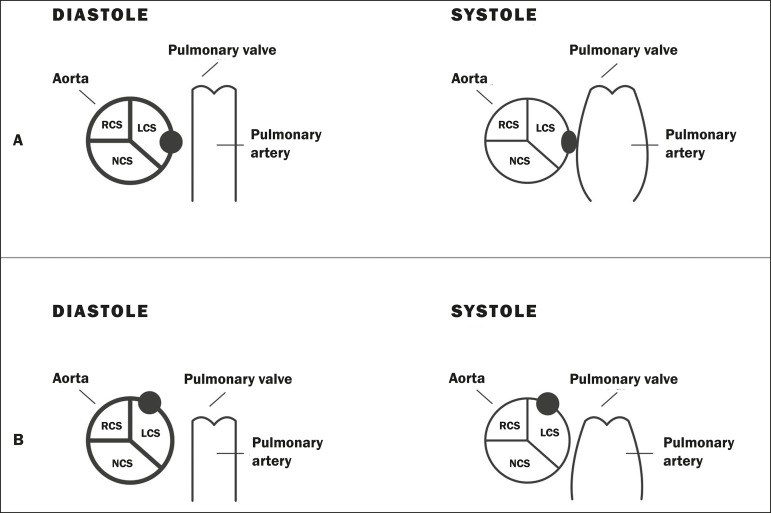
Illustration showing the anomalous interarterial course subtypes of the right coronary artery originating from the left coronary sinus. **A:** High interarterial course. The anomalous ostium (black ball in diastole) is located between the aorta and the pulmonary artery. During systole, there is simultaneous distension of the aorta and pulmonary artery, causing compression of the ostium (oval shape in systole). **B:** Low interarterial course. The anomalous ostium is located between the aorta and below the pulmonary valve. During systole, the aorta distends. However, when the right ventricle contracts, it does not compress the ostium (black ball in diastole and systole). RCS, right coronary sinus; LCS, left coronary sinus; NCS, noncoronary sinus.

When the RCA originates anomalously from the left coronary sinus and has an interarterial course, the height of its path in relation to the pulmonary valve can have different prognostic implications, with consequent stratification of risk and treatment. According to the finding on CT angiography, the interarterial course of the RCA is classified as either "high" or "low". A high course/outflow tract predisposes the patient to more adverse effects, such as angina and sudden death, and requires more attention on the part of the radiologist. This is due to the fact that, during systole, both vessels adjacent to the coronary artery (the aorta and pulmonary artery) dilate, narrowing the channel through which the anomalous coronary artery passes, a phenomenon that is aggravated during physical exercise. Conversely, when the interarterial course is low, the right ventricular outflow tract contracts during systole, counterbalancing the systolic expansion of the aorta and creating less narrowing in the coronary arterial course between the right ventricular outflow tract and the aorta^(^^8^^)^, as depicted in Figure 5.

## LCA

**Figure 6:**
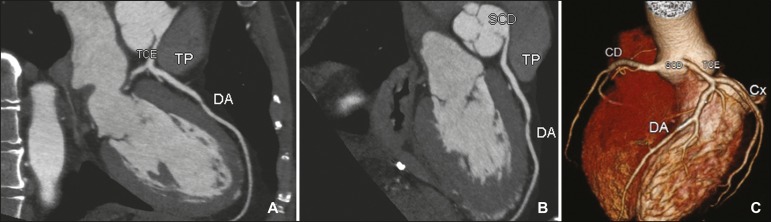
Contrast-enhanced CT. Coronal plane (**A**), oblique reconstruction (**B**), and a three-dimensional reconstruction (**C**) showing the anomalous origin of the trunk of the left coronary artery, adjacent to the origin of the right coronary artery in the right coronary sinus. The trunk of the left coronary artery branches to create the circumflex artery and the left anterior descending coronary artery. In these images, we can see the trajectory of the trunk of the left coronary artery passing behind the pulmonary trunk. TCE, trunk of the left coronary artery; TP, pulmonary trunk; DA, anterior descending coronary artery; CD, right coronary artery; Cx, circumflex coronary artery; SCD, right coronary sinus.

An interarterial course of the LCA can be seen in more than 75% of patients with an LCA originating from the right sinus (as a separate vessel or as a branch of a single coronary artery) (Figure 6). The high risk of sudden death is due to the acute angle of the ostium, the "stretching" of the intramural segment, and compression between the commissures of the right and left coronary cusps^(^^3^^)^. Sudden death can result from transient compression of the anomalous LCA course, caused by dilation of the aorta and pulmonary artery, which is in turn caused by the increase in blood flow during intense exercise, thus creating torsion or compression of the coronary artery between the aorta and the right ventricular outflow tract^(^^1^^)^.

## TREATMENT

Due to the difference in prognosis among these types of anomalies, the American College of Cardiology and the American Heart Association recommend surgical treatment for all patients with an LCA originating from the right sinus, regardless of symptoms or evidence of ischemia^(^^9^^)^. However, according to that same consensus, patients who present with an RCA originating from the left sinus should be submitted to surgical correction only if there is documented ischemia or significant symptomatology^(^^9^^)^.

## CONCLUSION

Coronary course anomalies can be observed on CT of the chest and CT angiography of the aorta or pulmonary arteries performed for different reasons. As such anomalies have variable prognoses, radiologists should be familiar with their presentations. A coronary anomaly with an interarterial course constitutes an important incidental finding that must be described and documented.
